# Editorial: Assessing and evaluating the impact of the COVID-19 pandemic on anxiety and stress: perspectives from North America

**DOI:** 10.3389/fpsyt.2023.1275415

**Published:** 2023-10-24

**Authors:** Mona Salehi, Man Amanat, Amir Garakani

**Affiliations:** ^1^Department of Psychiatry and Behavioral Health, Johns Hopkins University School of Medicine, Baltimore, MD, United States; ^2^Department of Neurology, Johns Hopkins University School of Medicine, Baltimore, MD, United States; ^3^Department of Psychiatry and Behavioral Health, Greenwich Hospital, Greenwich, CT, United States; ^4^Department of Psychiatry, Yale School of Medicine, New Haven, CT, United States

**Keywords:** COVID-19, anxiety, stress, United States, Canada

The COVID-19 pandemic significantly impacted the global mental health care system and the wellbeing of people. It had numerous potential effects on personal health, healthcare systems, the economy, and other areas previously unforeseen (1). During the COVID-19 pandemic, the development of strategic procedures toward enabling an environment for access to mental health services was challenging in most of the countries including Canada and the United States. The situation worsened due to increased demand for these services for anxiety and stress-related disorders (2, 3). In June 2020, the Centers for Disease Control and Prevention (CDC) conducted a nationwide survey involving over 5,000 US adults. The survey revealed that more than one-third of the respondents (40.9%) reported experiencing at least one adverse mental health condition, including depression and anxiety (4). A comprehensive systematic review assessed the impact of mental health issues throughout the COVID-19 pandemic. This review also highlighted a notably greater prevalence of anxiety and depression in North America in comparison to other geographical regions (5).

The objective of this Research Topic was to investigate the influence of the COVID-19 pandemic on anxiety and stress. The study aims to examine the impact of COVID-19 on the general population, patients, and healthcare workers (HCWs) in North America. The timeline includes both the initial wave as well as subsequent variants of COVID-19 and the effects of long COVID-19 in this region. This special issue includes five original research papers: three papers focusing on mental health in health care workers (HCW), one paper focusing on healthy adolescents, and one study with a special focus on mental health in post-acute sequelae of COVID-19.

The first paper on this issue is a mixed design study by Williams et al. which collected data from 457 physicians via self-report questionnaires. This study examines the impact of the COVID-19 pandemic on physician wellbeing, focusing on negative experiences and psychological distress in their personal and professional lives. Work system factors and positive interpersonal relationships were identified as key contributors and common challenges included the fear of contracting COVID-19 and dealing with distressed family members, while emotional support from family and friends emerged as a significant positive factor. The findings highlight the importance of targeted organizational and social support to alleviate pandemic-related stressors for physicians.

The original study done by Saravanan et al. investigated the occupational stress and burnout experienced by intensive care unit (ICU) nurses working with COVID and non-COVID patients. The research used a prospective longitudinal mixed-methods approach, following a cohort of 14 ICU nurses in a medical ICU (COVID unit) and 5 nurses in a cardiovascular ICU (non-COVID unit) for six 12-h shifts. Validated questionnaires and wrist-worn wearable technologies were utilized to collect data on stress and burnout prevalence and physiological indices of stress. Participants also provided insights on the causes of stress through open-ended questions. The findings, which were unsurprising, revealed that ICU nurses caring for COVID patients at the COVID unit were significantly more likely to experience stress compared to non-COVID unit nurses.

Cochran et al. assessed the sustained impact of the COVID-19 pandemic on the mental health of healthy adolescents. Fifteen adolescents completed self-report measures at three time points: pre-pandemic (T1), early pandemic (T2), and later pandemic (T3). These adolescents experienced sustained increases in depression and anxiety during the COVID-19 pandemic, which persisted from the early stage of the pandemic to later stages. Moreover, difficulties in emotion regulation during the early pandemic were linked to higher depression and anxiety levels at the later stage. These findings highlight the potential long-term mental health consequences for adolescents due to the pandemic.

Ferrando et al. investigated the prevalence and clinical characteristics of anxiety and post-traumatic stress symptoms in individuals recovering from COVID-19. This cohort consisted of 75 participants from a post-COVID-19 recovery program and the community. Clinically significant anxiety symptoms were found in 31% of the participants, and 29% exhibited symptoms of post-traumatic stress disorder (PTSD). Nervousness and excessive worry were the most common anxiety symptoms, while changes in mood/cognition and avoidance were prominent in PTSD. Comorbidity was observed between anxiety symptoms, PTSD, depression, and fatigue. Acute COVID-19 illness severity, prior psychiatric history, and memory complaints were predictive of clinically significant anxiety symptoms and PTSD. The study highlights the importance of screening for neuropsychiatric complications in individuals seeking care for post-acute sequelae of COVID-19, with specific attention to anxiety and PTSD symptoms.

The last longitudinal study by Gertler et al. examined the relationships between temperament traits, burnout, COVID concerns, moral injury, and mental health symptoms in 435 HCWs during the COVID-19 pandemic. Multidimensional Personality Questionnaire subscale scores for stress reaction and wellbeing were subjected to K-means cluster analyses and identified two groups of individuals: positive (low-stress reaction, high wellbeing) and negative (high-stress reaction, low wellbeing). Negative temperament individuals reported greater mood symptoms, burnout, and COVID concerns. Over time, their scores decreased, while positive temperament individuals' scores increased. Burnout played a significant role in mediating the group-by-time interaction, with the burnout exhaustion scale driving anxiety and depression symptoms. PTSD symptoms were also associated with COVID-19 health worries and negative temperament. Targeted interventions can reduce mood symptoms in negative-temperament individuals and prevent burnout in positive-temperament individuals during extended crises.

In conclusion, this themed collection enhances our knowledge of the negative effects of COVID-19 on mental health and highlights the importance of screening for neuropsychiatric complications of COVID-19 and developing strategies to screen and treat them in a multidisciplinary manner.

## Author contributions

MS: Conceptualization, Writing—original draft. MA: Writing—review & editing. AG: Writing—review & editing.
